# Supplementary material for: A new insight into underlying disease mechanism through semi-parametric latent differential network model
Source: BMC Bioinformatics. 2018 Dec 28;19(Suppl 17):493. doi: 10.1186/s12859-018-2461-2 (PMC6309076; doi:10.1186/s12859-018-2461-2)
Supplement: Supplementary file 1 — Contains the theoretical guarantee of of the proposed methods and proofs. (PDF 284 kb) [file 12859_2018_2461_MOESM1_ESM.pdf]

Supplement to "A new insight into underlying disease mechanism through semi-parametric latent differential network model"

Yong He[*], Jiadong Ji[†], Lei Xie[‡] Xinsheng Zhang[§] and Fuzhong Xue[¶]

Abstract

This supplementary materials contain the additional details of the paper "A new insight into underlying disease mechanism through semi-parametric latent differential network model" authored by Yong He, Jiadong JI, Xinsheng Zhang and Fuzhong Xue. Web Appendix A presents the technical conditions and theoretical results for the proposed estimators. Web Appendix B contains the proofs of the theorems in Web Appendix A.

Web Appendix A: Theoretical analysis

In Web Appendix A we will present theoretical analysis of the estimators $\widehat{\boldsymbol{\Delta}}$, $\widehat{\boldsymbol{\Delta}}^{\mathrm{B}}$ and $\widehat{\boldsymbol{\Delta}}^{\mathrm{M}}$ separately. We investigate the properties of the proposed estimators by considering the convergence rates of $\widehat{\boldsymbol{\Delta}}-\boldsymbol{\Delta}_0$, $\widehat{\boldsymbol{\Delta}}^{\mathrm{B}}-\boldsymbol{\Delta}_0^{\mathrm{B}}$ and $\widehat{\boldsymbol{\Delta}}^{\mathrm{M}}-\boldsymbol{\Delta}_0^{\mathrm{M}}$, including estimation error bounds and support recovery. Before we present the theoretical results, we first present some notations which are useful in the following sections. In this paper, notations $|\cdot|$ and $\|\cdot\|$ are used to denote element-wise norms and matrix norms respectively. For any vector $\boldsymbol{\mu}=(\mu_1,\ldots,\mu_d)\in R^d$, let $\boldsymbol{\mu}_{-i}$ denote the $(d-1)\times 1$ vector by removing the i-th entry from $\boldsymbol{\mu}$. $|\boldsymbol{\mu}|_0=\sum_{i=1}^d I(\mu_i\neq 0)$, $|\boldsymbol{\mu}|_1=\sum_{i=1}^d|\mu_i|$, $|\boldsymbol{\mu}|_2=\sqrt{\sum_{i=1}^d\mu_i^2}$ and $|\boldsymbol{\mu}|_\infty=\max_i|\mu_i|$. Let $\boldsymbol{A}=[a_{ij}]\in R^{d\times d}$. $\|\boldsymbol{A}\|_1=\max_{1\le j\le d}\sum_{i=1}^d|a_{ij}|$, $|\boldsymbol{A}|_\infty=\max_{i,j}|a_{ij}|$ and $|\boldsymbol{A}|_1=\sum_{i=1}^d\sum_{j=1}^d|a_{ij}|$. We use $\lambda_{\min}(\boldsymbol{A})$ and $\lambda_{\max}(\boldsymbol{A})$ to denote the smallest and largest eigenvalues of $\boldsymbol{A}$ respectively. $\mathrm{Vec}(\boldsymbol{A})$ denotes the $d^2\times 1$ vector obtained by stacking the columns of $\boldsymbol{A}$. For a set $\mathcal{H}$, we use $|\mathcal{H}|$ to denote the cardinality of $\mathcal{H}$. For two sequences of real numbers $\{a_n\}$ and $\{b_n\}$, we write $a_n=O(b_n)$ if there exists a constant C such that $|a_n|\le C|b_n|$ holds for all n, write $a_n=o(b_n)$ if $\lim_{n\to\infty}a_n/b_n=0$, and write $a_n\asymp b_n$ if there exist constants c and C such that $c\le a_n/b_n\le C$ for all n.

1 Theoretical properties for Gaussian copula model

First we introduce some basic conditions which are required to obtain the theoretical result for $\widehat{\boldsymbol{\Delta}}$. Condition (**C1**) requires the difference matrix to have essentially constant sparsity. This is reasonable in gene expression data analysis as it is expected that the underlying genetic networks share

[*]School of Statistics, Shandong University of Finance and Economics, China; e-mail: heyong@sdufe.edu.cn

[†]School of Statistics, Shandong University of Finance and Economics, China; e-mail: jiadong@sdufe.edu.cn

[‡]Ph.D. Program in Computer Science, The Graduate Center, The City University of New York,USA; Department of Computer Science, Hunter College, The City University of New York, USA; e-mail: lxie@iscb.org

[§]Department of Statistics, Management School, Fudan University, China;e-mail:xszhang@fudan.edu.cn

[¶]School of Public Health, Shandong University, China; e-mial:xuefzh@sdu.edu.cn

many common edges and do not differ much between two conditions. Condition (**C2**) requires that the covariates can not be too highly correlated, which is closely related to the mutual incoherence property introduce by Donoho and Huo (2001).

(**C1**) The true difference matrix $\mathbf{\Delta}_0$ has $s < p$ nonzero entries in its upper triangular part, and $|\mathbf{\Delta}_0|_1 \le M$, where M does not depend on p.

(**C2**) With s defined in Condition (**C1**), the constants $\sigma^X_{\max} = \max_{j\neq k} |\Sigma^X_{jk}|$ and $\sigma^Y_{\max} = \max_{j\neq k} |\Sigma^Y_{jk}|$ satisfy $\sigma = 4\max(\sigma^X_{\max}, \sigma^Y_{\max}) \le \sigma^P_{\min}(2s)^{-1}$, where $\sigma^P_{\min} = 2\min_{j,k}(1, 1 + \Sigma^Y_{kj}\Sigma^X_{jk})$.

Under these conditions, an additional thresholding step on the estimator $\widehat{\mathbf{\Delta}}$ with a careful chosen threshold leads to more accurate recovery of the differential network. Define the thresholded estimator

$$\widehat{\mathbf{\Delta}}_{\tau_n} = \{\widehat{\delta}_{jk} I(|\widehat{\delta}_{jk}| > \tau_n)\}.$$

Let $\widehat{\mathbf{\Delta}}_{\tau_n} = [\widehat{\delta}^{\tau_n}_{jk}]$ and define the function

$$\text{sgn}(t) = I(t > 0) - I(t < 0) + 0 \cdot I(t = 0).$$

Denote by $\mathcal{M}(\widehat{\mathbf{\Delta}}_{\tau_n}) = \{\text{sgn}(\widehat{\delta}^{\tau_n}_{jk}) : j = 1, \ldots, p; k = 1, \ldots, p\}$ and $\mathcal{M}(\mathbf{\Delta}_0) = \{\text{sgn}(\delta^0_{jk}) : j = 1, \ldots, p; k = 1, \ldots, p\}$ the vectors of the signs of the entries of the estimated and true difference matrices, respectively. The following theorem establishes that $\widehat{\mathbf{\Delta}}_{\tau_n}$ can recover not only the support of $\mathbf{\Delta}_0$ but also the signs of its nonzero entries as long as those entries are sufficiently large.

Theorem 1.1. Assume that conditions (**C1**) and (**C2**) hold. If $\min(n_X, n_Y) > \log p$,

$$\tau_n \ge \frac{2}{\sigma^P_{\min}} \left\{ 1 + \frac{\sigma^P_{\min}}{\sigma^P_{\min} - (2s-1)\sigma} \right\} C \sqrt{\frac{\log p}{\min(n_X, n_Y)}},$$

and $\min_{j,k:\delta^0_{jk}\neq 0} |\delta^0_{jk}| > 2\tau_n$, then $\mathcal{M}(\widehat{\mathbf{\Delta}}_{\tau_n}) = \mathcal{M}(\widehat{\mathbf{\Delta}}_0)$ with probability at least $1 - p^{-1}$, where C is a sufficiently large constant independent of (p, n_X, n_Y) and $\sigma^P_{\min}, \sigma$ are defined in condition (**C2**).

In the context of genetic networks, Theorem 1.1 guarantees that $\widehat{\mathbf{\Delta}}_{\tau_n}$ can identify genes whose conditional dependencies change in magnitude as well as the directions of those changes between two conditions under certain regularity conditions. The τ_n is tuning parameter which is set to be 10^{-4} in the simulation and data analysis.

The following theorem establishes the convergence rate of $\widehat{\mathbf{\Delta}} - \mathbf{\Delta}_0$ in the Frobenius norm.

Theorem 1.2. Suppose that conditions (**C1**) and (**C2**) hold. If $\min(n_X, n_Y) > \log p$,

$$\lambda_n = C\sqrt{\frac{\log p}{\min(n_X, n_Y)}},$$

then

$$\|\widehat{\mathbf{\Delta}} - \mathbf{\Delta}_0\|_{\text{F}} \le \frac{2\sqrt{5s}}{\sigma^P_{\min}} \left\{ 1 + \frac{\sigma^P_{\min}}{\sigma^P_{\min} - (2s-1)\sigma} \right\} \lambda_n$$

with probability at least $1-2p^{-2}$, where C is a sufficiently large constant independent of (p, n_X, n_Y) and $\sigma^P_{\min}, \sigma$ are defined in condition (**C2**).

The following theorem establishes the elementwise ℓ_∞ norm bound of the estimation error, which is critical in the proof of Theorem 1.1 and 1.2.

Theorem 1.3. Suppose that conditions (**C1**) and (**C2**) hold. If $\min(n_X, n_Y) > \log p$,

$$\lambda_n = C\sqrt{\frac{\log p}{\min(n_X, n_Y)}},$$

then

$$|\widehat{\mathbf{\Delta}} - \mathbf{\Delta}_0|_\infty \leq \frac{2}{\sigma^P_{\min}}\left\{1 + \frac{\sigma^P_{\min}}{\sigma^P_{\min} - (2s-1)\sigma}\right\}\lambda_n$$

with probability at least $1-2p^{-2}$, where C is a sufficiently large constant independent of (p, n_X, n_Y) and $\sigma^P_{\min}, \sigma$ are defined in condition (**C2**).

The same parametric convergence rate for differential network under Gaussian assumption was established by Zhao et al. (2014). This shows that the estimator $\widehat{\mathbf{\Delta}}$ for Gaussian copula model achieves the optimal parametric rate $\sqrt{\log p/\min(n_X, n_Y)}$ in terms of difference matrix estimation. The extra modeling flexibility and robustness come at almost no cost of statistical efficiency. Thus this new estimator can be used as a safe replacement of Gaussian estimators even when the data are truly Gaussian. This is one main contribution of the current paper.

2 Theoretical properties for latent Gaussian copula model for binary data

First we introduce some basic conditions which is required to obtain the theoretical results for binary data. Conditions **(B1)** and **(B2)** are similar conditions to conditions **(C1)** and **(C2)** in the last section. Conditions **(B3)** and **(B4)** are mainly adopted for technical considerations and impose little restriction in practice. Specifically, Condition **(B3)** rules out the singular case that there exist variables which are perfectly collinear. Condition **(B4)** is used to control the variation of $F^{-1}(\tau; \Lambda_j, \Lambda_k)$ with respect to $(\tau; \Lambda_j, \Lambda_k)$.

(**B1**) The true difference matrix $\mathbf{\Delta}^{\mathrm{B}}_0$ has $s < p$ nonzero entries in its upper triangular part, and $|\mathbf{\Delta}^{\mathrm{B}}_0|_1 \leq M$, where M does not depend on p.

(**B2**) With s defined in Condition (**B1**), the constants $\sigma^1_{\max} = \max_{j\neq k}|\Sigma^1_{jk}|$ and $\sigma^2_{\max} = \max_{j\neq k}|\Sigma^2_{jk}|$ satisfy $\sigma = 4\max(\sigma^1_{\max}, \sigma^2_{\max}) \leq \sigma^P_{\min}(2s)^{-1}$, where $\sigma^P_{\min} = 2\min_{j,k}(1, 1+\Sigma^2_{kj}\Sigma^1_{jk})$.

(**B3**) There exist a constant δ such that $\max\{\Sigma^1_{jk}, \Sigma^2_{jk}\} \leq 1-\delta$ for any $1 \leq j \neq k \leq p$.

(**B4**) There exists a constant $M > 0$ such that $\max\{|\Lambda^1_j|, |\Lambda^2_j|\} \leq M$ for any $1 \leq j \leq p$.

Under these conditions, an additional thresholding step on the estimator $\widehat{\mathbf{\Delta}}^{\mathrm{B}}$ with a careful chosen threshold leads to more accurate recovery of the differential network. Define the thresholded estimator

$$\widehat{\mathbf{\Delta}}^{\mathrm{B}}_{\tau_n} = \{\widehat{\delta}^{\mathrm{B}}_{jk} I(|\widehat{\delta}^{\mathrm{B}}_{jk}| > \tau_n)\}.$$

Denote by $\mathcal{M}(\widehat{\mathbf{\Delta}}^{\mathrm{B}}_{\tau_n}) = \{\mathrm{sgn}(\widehat{\delta}^{\tau_n\mathrm{B}}_{jk}) : j = 1,\ldots,p; k = 1,\ldots,p\}$ and $\mathcal{M}(\mathbf{\Delta}^{\mathrm{B}}_0) = \{\mathrm{sgn}(\delta^{0\mathrm{B}}_{jk}) : j = 1,\ldots,p; k = 1,\ldots,p\}$ the vectors of the signs of the entries of the estimated and true difference matrices, respectively. The following theorem establishes that $\widehat{\mathbf{\Delta}}^{\mathrm{B}}_{\tau_n}$ can recover not only the support of $\mathbf{\Delta}^{\mathrm{B}}_0$ but also the signs of its nonzero entries as long as those entries are sufficiently large.

Theorem 2.1. Assume that conditions (**B1**)—(**B4**) hold. If $\min(n_1, n_2) > \log p$,

$$\tau_n \geq \frac{2}{\sigma^P_{\min}} \left\{1 + \frac{\sigma^P_{\min}}{\sigma^P_{\min} - (2s-1)\sigma}\right\} C\sqrt{\frac{\log p}{\min(n_1,n_2)}},$$

and $\min_{j,k:\delta^{0\mathrm{B}}_{jk}\neq 0} |\delta^{0\mathrm{B}}_{jk}| > 2\tau_n$, then $\mathcal{M}(\widehat{\mathbf{\Delta}}^{\mathrm{B}}_{\tau_n}) = \mathcal{M}(\mathbf{\Delta}^{\mathrm{B}}_0)$ with probability at least $1 - p^{-1}$, where C is a sufficiently large constant independent of (p, n_1, n_2) and $\sigma^P_{\min}, \sigma$ are defined in condition (**B2**).

In the context of genetic networks, Theorem 2.1 guarantees that $\widehat{\mathbf{\Delta}}^{\mathrm{B}}_{\tau_n}$ can identify genes whose conditional dependencies change in magnitude as well as the directions of those changes between two conditions under certain regularity conditions even if we only see the binary data 0/1. The τ_n is a tuning parameter which is set to be 10^{-4} in the simulation and data analysis.

The following theorem establishes the convergence rate of $\widehat{\mathbf{\Delta}}^{\mathrm{B}} - \mathbf{\Delta}^{\mathrm{B}}_0$ in the Frobenius norm.

Theorem 2.2. Suppose that conditions (**B1**)—(**B4**) hold. If $\min(n_1, n_2) > \log p$,

$$\lambda_n = C\sqrt{\frac{\log p}{\min(n_1,n_2)}},$$

then

$$\|\widehat{\mathbf{\Delta}}^{\mathrm{B}} - \mathbf{\Delta}^{\mathrm{B}}_0\|_{\mathrm{F}} \leq \frac{2\sqrt{5s}}{\sigma^P_{\min}} \left\{1 + \frac{\sigma^P_{\min}}{\sigma^P_{\min} - (2s-1)\sigma}\right\} \lambda_n$$

with probability at least $1 - p^{-1}$, where C is a sufficiently large constant independent of (p, n_1, n_2) and $\sigma^P_{\min}, \sigma$ are defined in condition (**B2**).

The following theorem establishes the elementwise ℓ_∞ norm bound of the estimation error, which is critical in the proof of Theorem 2.2 and 2.3.

Theorem 2.3. Suppose that conditions (**B1**)—(**B4**) hold. If $\min(n_1, n_2) > \log p$,

$$\lambda_n = C\sqrt{\frac{\log p}{\min(n_1,n_2)}},$$

then

$$|\widehat{\mathbf{\Delta}}^{\mathrm{B}} - \mathbf{\Delta}^{\mathrm{B}}_0|_\infty \leq \frac{2}{\sigma^P_{\min}} \left\{1 + \frac{\sigma^P_{\min}}{\sigma^P_{\min} - (2s-1)\sigma}\right\} \lambda_n$$

with probability at least $1 - p^{-1}$, where C is a sufficiently large constant independent of (p, n_1, n_2) and $\sigma^P_{\min}, \sigma$ are defined in condition (**C2**).

Theorem 2.1 — Theorem 2.3 establish that the proposed differential network estimator $\widehat{\mathbf{\Delta}}^{\mathrm{B}}$ achieves the same rate of convergence for both matrix estimation and graph recovery, as if the latent Gaussian copula random variable $\boldsymbol{X}$ were observed.

3 Theoretical properties for latent Gaussian copula model for mixed data

First we introduce some basic conditions which is required to obtain the theoretical results for mixed data. Conditions **(M1)** and **(M2)** are similar to conditions **(C1)** and **(C2)** in Section 1. Conditions **(M3)** and **(M4)** are similar to conditions **(B1)** and **(B2)** in Section 2.

(**M1**) The true difference matrix $\boldsymbol{\Delta}_0^{\mathrm{M}}$ has $s < p$ nonzero entries in its upper triangular part, and $|\boldsymbol{\Delta}_0^{\mathrm{M}}|_1 \leq M$, where M does not depend on p.

(**M2**) With s defined in Condition (**M1**), the constants $\sigma_{\max}^1 = \max_{j \neq k} |\Sigma_{jk}^1|$ and $\sigma_{\max}^2 = \max_{j \neq k} |\Sigma_{jk}^2|$ satisfy $\sigma = 4\max(\sigma_{\max}^1, \sigma_{\max}^2) \leq \sigma_{\min}^P (2s)^{-1}$, where $\sigma_{\min}^P = 2\min_{j,k}(1, 1 + \Sigma_{kj}^2 \Sigma_{jk}^1)$.

(**M3**) There exist a constant δ such that $\max\{\Sigma_{jk}^1, \Sigma_{jk}^2\} \leq 1 - \delta$ for any $1 \leq j \neq k \leq p_1$.

(**M4**) There exists a constant $M > 0$ such that $\max\{|\Lambda_j^1|, |\Lambda_j^2|\} \leq M$ for any $1 \leq j \leq p_1$.

Under these conditions, an additional thresholding step on the estimator $\widehat{\boldsymbol{\Delta}}^{\mathrm{M}}$ with a careful chosen threshold leads to more accurate recovery of the differential network. Define the thresholded estimator

$$\widehat{\boldsymbol{\Delta}}_{\tau_n}^{\mathrm{M}} = \{\widehat{\delta}_{jk}^{\mathrm{M}} I(|\widehat{\delta}_{jk}^{\mathrm{M}}| > \tau_n)\}.$$

Denote by $\mathcal{M}(\widehat{\boldsymbol{\Delta}}_{\tau_n}^{\mathrm{M}}) = \{\mathrm{sgn}(\widehat{\delta}_{jk}^{\tau_n \mathrm{B}}) : j = 1, \ldots, p; k = 1, \ldots, p\}$ and $\mathcal{M}(\boldsymbol{\Delta}_0^{\mathrm{M}}) = \{\mathrm{sgn}(\delta_{jk}^{0\mathrm{M}}) : j = 1, \ldots, p; k = 1, \ldots, p\}$ the vectors of the signs of the entries of the estimated and true difference matrices, respectively. The following theorem establishes that $\widehat{\boldsymbol{\Delta}}_{\tau_n}^{\mathrm{M}}$ can recover not only the support of $\boldsymbol{\Delta}_0^{\mathrm{M}}$ but also the signs of its nonzero entries as long as those entries are sufficiently large.

Theorem 3.1. Assume that conditions (**M1**)—(**M4**) hold. If $\min(n_1, n_2) > \log p$,

$$\tau_n \geq \frac{2}{\sigma_{\min}^P}\left\{1 + \frac{\sigma_{\min}^P}{\sigma_{\min}^P - (2s-1)\sigma}\right\} C \sqrt{\frac{\log p}{\min(n_1, n_2)}},$$

and $\min_{j,k:\delta_{jk}^{0\mathrm{M}} \neq 0} |\delta_{jk}^{0\mathrm{M}}| > 2\tau_n$, then $\mathcal{M}(\widehat{\boldsymbol{\Delta}}_{\tau_n}^{\mathrm{M}}) = \mathcal{M}(\boldsymbol{\Delta}_0^{\mathrm{M}})$ with probability at least $1 - p^{-1}$, where C is a sufficiently large constant independent of (p, n_1, n_2) and $\sigma_{\min}^P, \sigma$ are defined in condition (**M2**).

The following theorem establishes the convergence rate of $\widehat{\boldsymbol{\Delta}}^{\mathrm{M}} - \boldsymbol{\Delta}_0^{\mathrm{M}}$ in the Frobenius norm.

Theorem 3.2. Suppose that conditions (**M1**)—(**M4**) hold. If $\min(n_1, n_2) > \log p$,

$$\lambda_n = C\sqrt{\frac{\log p}{\min(n_1, n_2)}},$$

then

$$\|\widehat{\boldsymbol{\Delta}}^{\mathrm{M}} - \boldsymbol{\Delta}_0^{\mathrm{M}}\|_{\mathrm{F}} \leq \frac{2\sqrt{5s}}{\sigma_{\min}^P}\left\{1 + \frac{\sigma_{\min}^P}{\sigma_{\min}^P - (2s-1)\sigma}\right\} \lambda_n$$

with probability at least $1-p^{-1}$, where C is a sufficiently large constant independent of (p, n_1, n_2) and $\sigma^P_{\min}, \sigma$ are defined in condition (**M2**).

The following theorem establishes the elementwise ℓ_∞ norm bound of the estimation error, which is critical in the proof of Theorem 3.1 and 3.2.

Theorem 3.3. Suppose that conditions (**M1**)—(**M4**) hold. If $\min(n_1, n_2) > \log p$,

$$\lambda_n = C\sqrt{\frac{\log p}{\min(n_1, n_2)}},$$

then

$$|\widehat{\mathbf{\Delta}}^{\mathrm{M}} - \mathbf{\Delta}_0^{\mathrm{M}}|_\infty \le \frac{2}{\sigma^P_{\min}}\left\{1 + \frac{\sigma^P_{\min}}{\sigma^P_{\min} - (2s-1)\sigma}\right\}\lambda_n$$

with probability at least $1-p^{-1}$, where C is a sufficiently large constant independent of (p, n_1, n_2) and $\sigma^P_{\min}, \sigma$ are defined in condition (**M2**).

Theorem 3.1 — Theorem 3.3 establish that the proposed differential network estimator $\widehat{\mathbf{\Delta}}^{\mathrm{M}}$ achieves the same parametric rate of convergence for both matrix estimation and graph recovery.

Remark 3.4. Compared to the separate and joint approaches to estimating differential networks Cai et al. (2011); Guo et al. (2011) which require sparsity on each $\mathbf{\Sigma}^{-1}$, the proposed direction estimation methods for different types of data only require the sparsity of the difference matrix $\mathbf{\Delta}_0$. Thus the theoretical results in Theorem 1.1-3.3 can still hold in the presence of hub nodes.

Web Appendix B: Proofs of Main Theorems

Before we give the detailed proofs of main theorems in Web Appendix A, we first present some useful lemmas. Lemma 3.5 is established in Zhao et al. (2014).

Lemma 3.5. Let $\mathbf{\Sigma} = \mathbf{\Sigma}^Y \otimes \mathbf{\Sigma}^X$. Label the entries of $\mathbf{\Gamma}^\top\mathbf{\Sigma}\mathbf{\Gamma}$ as $\Sigma^\Gamma_{j'k',jk}$ ($1 \le j' \le k' \le p$; $1 \le j \le k \le p$). Then we have

$$\begin{array}{lll}
\Sigma^\Gamma_{j'k',jk} & = \Sigma^Y_{k'k}\Sigma^X_{j'j} + \Sigma^Y_{k'j}\Sigma^X_{j'k} + \Sigma^Y_{j'k}\Sigma^X_{k'j} + \Sigma^Y_{j'j}\Sigma^X_{k'k}, & j' \ne k', j \ne k; \\
\Sigma^\Gamma_{j'k',jj} & = \Sigma^Y_{k'j}\Sigma^X_{j'j} + \Sigma^Y_{j'j}\Sigma^X_{k'j}, & j' \ne k', j = k; \\
\Sigma^\Gamma_{j'j',jk} & = \Sigma^Y_{j'k}\Sigma^X_{j'j} + \Sigma^Y_{j'j}\Sigma^X_{j'k}, & j' = k', j \ne k; \\
\Sigma^\Gamma_{j'j',jj} & = \Sigma^Y_{j'j}\Sigma^X_{j'j}, & j' = k', j = k;
\end{array}$$

Lemma 3.6. With probability at least $1 - 2/p^2$, we have

$$\sup_{j,k}\left|\widehat{S}^X_{jk} - \Sigma^X_{jk}\right| \le C\sqrt{\frac{\log p}{n_X}}, \quad \sup_{j,k}\left|\widehat{S}^Y_{jk} - \Sigma^Y_{jk}\right| \le C\sqrt{\frac{\log p}{n_Y}} \tag{3.1}$$

where $\widehat{S}^X_{jk}, \widehat{S}^Y_{jk}$ are defined in Equation (2.1) in the main paper and C is a constant independent of (n_X, n_Y, p).

Proof. We prove the result for $\widehat{S}^X_{jk}$. The result for $\widehat{S}^Y_{jk}$ can be obtained in a similar way. By definition,

$$\widehat{S}^X_{jk} = \begin{cases} \sin(\frac{\pi}{2}\widehat{\tau}^X_{jk}) & j \neq k \\ 1 & j = k \end{cases}.$$

Denote by $\boldsymbol{T}^X = [\tau^X_{jk}]$ and $\widehat{\boldsymbol{T}}^X = [\widehat{\tau}^X_{jk}]$. By Taylor expansion, we have

$$\widehat{S}^X_{jk} - \Sigma^X_{jk} = \frac{\pi}{2}\cos(\frac{\pi}{2}\xi_{jk})(\widehat{\tau}^X_{jk} - \tau^X_{jk}),$$

where $\xi_{jk} \in \Big[\min\{\widehat{\tau}^X_{jk}, \tau^X_{jk}\}, \max\{\widehat{\tau}^X_{jk}, \tau^X_{jk}\}\Big]$. Denote by $\widetilde{\boldsymbol{T}}^X = [\xi_{jk}]$, then we have

$$\widehat{\boldsymbol{S}}^X - \boldsymbol{\Sigma}^X = \frac{\pi}{2}\cos(\frac{\pi}{2}\widetilde{\boldsymbol{T}}^X) \circ (\widehat{\boldsymbol{T}}^X - \boldsymbol{T}^X),$$

where $\circ$ denotes the matrix element-wise multiplication, which implies that

$$|\widehat{\boldsymbol{S}}^X - \boldsymbol{\Sigma}^X|_\infty \leq \frac{\pi}{2}|\widehat{\boldsymbol{T}}^X - \boldsymbol{T}^X|_\infty.$$

By Hoeffding inequality, we have that

$$P\left(|\widehat{\tau}^X_{jk} - \tau^X_{jk}| > t\right) \leq 2\exp(-n_X t^2/4).$$

Therefore,

$$P\left(|\widehat{\boldsymbol{T}}^X - \boldsymbol{T}^X|_\infty > t\right) \leq 2p^2\exp(-n_X t^2/4).$$

By letting $t = 4\sqrt{\log p/n_X}$, the above inequality implies that with probability $1 - 2p^{-2}$,

$$|\widehat{\boldsymbol{T}}^X - \boldsymbol{T}^X|_\infty \leq C\sqrt{\frac{\log p}{n_X}}.$$

Thus with probability at least $1 - 2p^{-2}$,

$$|\widehat{\boldsymbol{S}}^X - \boldsymbol{\Sigma}^X|_\infty \leq \frac{\pi}{2}|\widehat{\boldsymbol{T}}^X - \boldsymbol{T}^X|_\infty \leq C_1\sqrt{\frac{\log p}{n_X}},$$

where $C_1 = \pi/2C$. This concludes Lemma 3.6. □

Lemma 3.7. Suppose that conditions **(B3)** and **(B4)** hold. With probability at least $1 - 1/p$, we have

$$\sup_{j,k}\left|\widehat{\mathrm{R}}^1_{jk} - \Sigma^1_{jk}\right| \leq C\sqrt{\frac{\log p}{n_1}}, \quad \sup_{j,k}\left|\widehat{\mathrm{R}}^1_{jk} - \Sigma^2_{jk}\right| \leq C\sqrt{\frac{\log p}{n_2}}, \tag{3.2}$$

where $\widehat{R}^1_{jk}, \widehat{R}^2_{jk}$ are defined in Equation (2.5) and (2.6) in the main paper and C is a constant independent of (n_1, n_2, p).

Proof. We prove the result for $\widehat{\mathrm{R}}^1_{jk}$. The result for $\widehat{\mathrm{R}}^2_{jk}$ can be obtained in a similar way. By the argument in Fan et al. (2017), we have that for any $t > 0$,

$$\begin{aligned} P\left(|\widehat{\mathbf{R}}^1 - \boldsymbol{\Sigma}^1|_\infty > t\right) \leq & 2p^2\exp\left(-\frac{n_1 t^2}{8L_2^2}\right) + 4p^2\exp\left(-\frac{n_1 t^2\pi}{16^2 L_1^2 L_2^2}\right) \\ & + 4p^2\exp\left(-\frac{M^2 n_1}{2L_1^2}\right), \end{aligned}$$

where L_1, L_2 are two positive constants independent of (n_1, p). Thus let $t = C\sqrt{\frac{\log p}{n_1}}$ with a sufficiently large constant C, we have

$$\sup_{j,k}\left|\widehat{\mathrm{R}}_{jk}^1 - \Sigma_{jk}^1\right| \leq C\sqrt{\frac{\log p}{n_1}}.$$

□

Lemma 3.8. Fan et al. (2017) Suppose that conditions **(M3)** and (**M4**) hold. With probability greater than $1 - 1/p$, we have that

$$\sup_{j,k}\left|\widehat{\mathrm{T}}_{jk}^1 - \Sigma_{jk}^1\right| \leq C\sqrt{\frac{\log p}{n_1}}, \quad \sup_{j,k}\left|\widehat{\mathrm{T}}_{jk}^1 - \Sigma_{jk}^2\right| \leq C\sqrt{\frac{\log p}{n_2}}, \tag{3.3}$$

where $\widehat{T}_{jk}^1, \widehat{T}_{jk}^2$ are defined in Equation (2.8), (2.9) and (2.10) in the main paper and C is a constant independent of (n_1, n_2, p).

Proof of Theorem 1.3

Let the entries of $\mathbf{\Delta}_0$ be denoted by δ_{jk}^0 and define the $p(p+1)/2 \times 1$ vector $\boldsymbol{\theta}_0 = (\delta_{jk}^0)_{1\leq j\leq k\leq p}$. Define $\mathbf{\Sigma} = \mathbf{\Sigma}^Y \otimes \mathbf{\Sigma}^X$. Label the entries of $\mathbf{\Gamma}^\top\mathbf{\Sigma}\mathbf{\Gamma}$ as $\Sigma_{j'k',jk}^\Gamma (1 \leq j' \leq k' \leq p;\ 1 \leq j \leq k \leq p$). Let $\widehat{\boldsymbol{S}} = \widehat{\boldsymbol{S}}^X \otimes \widehat{\boldsymbol{S}}^Y$, $\widehat{\boldsymbol{s}} = \mathrm{Vec}(\widehat{\boldsymbol{S}}^X - \widehat{\boldsymbol{S}}^Y)$, $\boldsymbol{s} = \mathrm{Vec}(\mathbf{\Sigma}^X - \mathbf{\Sigma}^Y)$ and $\boldsymbol{w} = \widehat{\boldsymbol{\theta}} - \boldsymbol{\theta}_0$. The bound on $|\widehat{\mathbf{\Delta}} - \mathbf{\Delta}_0|_\infty = |\boldsymbol{w}|_\infty$ is obtained by following the similar argument as in Zhao et al. (2014).

Denote the ath component of $\mathbf{\Gamma}^\top\mathbf{\Sigma}\mathbf{\Gamma}\boldsymbol{w}$ by $(\mathbf{\Gamma}^\top\mathbf{\Sigma}\mathbf{\Gamma}\boldsymbol{w})_a$, the (a,b)th entry of $\mathbf{\Gamma}^\top\mathbf{\Sigma}\mathbf{\Gamma}$ by Σ_{ab}^Γ, and the bth component of $\boldsymbol{w}$ by w_b. Denote by $\Sigma_{\max}^\Gamma = \max_{a\neq b}|\Sigma_{ab}^\Gamma|$. Then we have that

$$(\mathbf{\Gamma}^\top\mathbf{\Sigma}\mathbf{\Gamma}\boldsymbol{w})_a = \sum_b \Sigma_{ab}^\Gamma w_b = \Sigma_{aa}^\Gamma w_a + \sum_{b\neq a}\Sigma_{ab}^\Gamma w_b,$$

which further implies that

$$|\Sigma_{aa}^\Gamma w_a| \leq |\mathbf{\Gamma}^\top\mathbf{\Sigma}\mathbf{\Gamma}\boldsymbol{w}|_\infty + \Sigma_{\max}^\Gamma\sum_{b\neq a}|w_b|. \tag{3.4}$$

The diagonal terms Σ_{aa}^Γ can be labeled as $\Sigma_{jk,jk}^\Gamma$, where j may equal to k. By Lemma 3.5, we have that $\Sigma_{jk,jk}^\Gamma \geq \sigma_{\min}^P$, with $\sigma_{\min}^P$ define in condition (**C2**). The off-diagonal terms $\Sigma_{ab}^\Gamma, a \neq b$, can be relabelled as $\Sigma_{j'k',jk}^\Gamma$ with $j' \neq j$ or $k' \neq k$. By Lemma 3.5, we have that $\Sigma_{j'k',jk}^\Gamma \leq 4\max(\sigma_{\max}^X, \sigma_{\max}^Y) = \sigma$, with $\sigma_{\max}^X, \sigma_{\max}^Y$ defined as in condition condition (**C2**). By these facts and together with condition (**C2**), Equation (3.4) becomes

$$|\boldsymbol{w}|_\infty \leq \frac{1}{\sigma_{\min}^P}\left(|\mathbf{\Gamma}^\top\mathbf{\Sigma}\mathbf{\Gamma}\boldsymbol{w}| + \frac{\sigma_{\min}^P}{2s}|\boldsymbol{w}|_1\right). \tag{3.5}$$

Let $\boldsymbol{\theta}_0 = (\theta_{01}, \ldots, \theta_{0d})^\top$ and $Q_0 = \{1 \leq i \leq d : \theta_{0i} \neq 0\}$, where $d = p(p+1)/2$. For any $d \times 1$ vector $\boldsymbol{a} = (a_1, \ldots, a_d)^\top$, let $\boldsymbol{a}_{Q_0}$ be the vector with component $a_{Q_0j} = 0$ for $j \neq Q_0$ and $a_{Q_0j} = a_j$ for $j \in Q_0$.

First we will show that $\boldsymbol{\theta}_0$ is the in the feasible set in Equation (2.12) in the main paper with high probability. By Lemma 3.6, we have that both $|\widehat{\boldsymbol{S}}^X - \boldsymbol{\Sigma}^X|_\infty$ and $|\widehat{\boldsymbol{S}}^Y - \boldsymbol{\Sigma}^Y|_\infty$ are less than $C\left(\log p/\min(n_X, n_Y)\right)^{1/2}$ with probability at least $1 - 2p^{-2}$. Thus

$$\begin{aligned}
|\boldsymbol{\Gamma}^\top \widehat{\boldsymbol{S}} \boldsymbol{\Gamma} \boldsymbol{\theta}_0 - \boldsymbol{\Gamma}^\top \widehat{\boldsymbol{s}}|_\infty &\le |\boldsymbol{\Gamma}^\top (\widehat{\boldsymbol{S}} - \boldsymbol{\Sigma}) \boldsymbol{\Gamma} \boldsymbol{\theta}_0|_\infty + |\boldsymbol{\Gamma}^\top (\widehat{\boldsymbol{s}} - \boldsymbol{s})|_\infty \\
&\le \|\boldsymbol{\Gamma}^\top\|_\infty |\widehat{\boldsymbol{S}} - \boldsymbol{\Sigma}|_\infty \|\boldsymbol{\Gamma}\|_1 |\boldsymbol{\theta}_0|_1 \\
&\quad + \|\boldsymbol{\Gamma}^\top\|_\infty \left(|\widehat{\boldsymbol{S}}^X - \boldsymbol{\Sigma}^X|_\infty + |\widehat{\boldsymbol{S}}^Y - \boldsymbol{\Sigma}^Y|_\infty \right) \\
&\le 4M |\widehat{\boldsymbol{S}} - \boldsymbol{\Sigma}|_\infty + 4C\left(\log p/\min(n_X, n_Y)\right)^{1/2}
\end{aligned}$$

where $\|\boldsymbol{\Gamma}\|_1 = 2$ by the definition of $\boldsymbol{\Gamma}$ and $|\boldsymbol{\theta}_0|_1 \le M$. By the definition of the Kronecker product, each entry of $\boldsymbol{\Sigma}$ can be written as $\Sigma^X_{l'l}\Sigma^Y_{m'm}$, so

$$\begin{aligned}
&|\widehat{S}^X_{l'l}\widehat{S}^Y_{m'm} - \Sigma^X_{l'l}\Sigma^Y_{m'm}| \\
&= |\Sigma^X_{l'l}(\widehat{S}^Y_{m'm} - \Sigma^Y_{m'm}) + (\widehat{S}^X_{l'l} - \Sigma^X_{l'l})\Sigma^Y_{m'm} + (\widehat{S}^X_{l'l} - \Sigma^X_{l'l})(\widehat{S}^Y_{m'm} - \Sigma^Y_{m'm})| \\
&\le \left[|\Sigma^X_{l'l}| + |\Sigma^Y_{m'm}| + C\left(\log p/\min(n_X, n_Y)\right)^{1/2} \right] C\left(\log p/\min(n_X, n_Y)\right)^{1/2} \\
&\le [2 + C]\, C\left(\log p/\min(n_X, n_Y)\right)^{1/2} \\
&\le C_1 \left(\log p/\min(n_X, n_Y)\right)^{1/2},
\end{aligned}$$

since $\min(n_X, n_Y) > \log p$. Then $\boldsymbol{\theta}_0$ is feasible with probability at least $1 - 2p^{-2}$ if $\lambda_n = C\left(\log p/\min(n_X, n_Y)\right)^{1/2}$ with a sufficiently large constant C.

Next we will give an bound on $|\boldsymbol{w}|_1$. By the definition of $\widehat{\boldsymbol{\theta}}$ in Equation (2.12) in the main paper, we have $|\boldsymbol{\theta}_0|_1 \ge |\widehat{\boldsymbol{\theta}}|_1$, which implies that $|\boldsymbol{\theta}_{0Q_0}|_1 - (|\widehat{\boldsymbol{\theta}}_{Q_0}|_1 + |\widehat{\boldsymbol{\theta}}_{Q_0^c}|_1) \ge 0$. By triangle inequality, $|\boldsymbol{\theta}_{0Q_0} - \widehat{\boldsymbol{\theta}}_{Q_0}|_1 \ge |\widehat{\boldsymbol{\theta}}_{Q_0^c}|_1$, or in other words, $|\boldsymbol{w}_{Q_0^c}|_1 \le |\boldsymbol{w}_{Q_0}|_1$. Therefore, we have $|\boldsymbol{w}|_1 \le 2|\boldsymbol{w}_{Q_0}|_1 \le 2s^{1/2}|\boldsymbol{w}_{Q_0}|_2$. To bound $|\boldsymbol{w}_{Q_0}|_2$, we have that for any s-sparse vector $\boldsymbol{c}$,

$$\begin{aligned}
|\boldsymbol{c}^\top \boldsymbol{\Gamma}^\top \boldsymbol{\Sigma} \boldsymbol{\Gamma} \boldsymbol{c}| &\ge \sum_a \Sigma^\Gamma_{aa} c_a^2 - \left| \sum_{a \ne b} \Sigma^\Gamma_{ab} c_a c_b \right| \\
&\ge \sigma^P_{\min} |\boldsymbol{c}|_2^2 - \sigma \sum_{a \ne b} |c_a c_b| \\
&\ge \sigma^P_{\min} |\boldsymbol{c}|_2^2 - \sigma(s-1)|\boldsymbol{c}|_2^2,
\end{aligned}$$

which implies that

$$\begin{aligned}
|\boldsymbol{w}_{Q_0}^\top \boldsymbol{\Gamma}^\top \boldsymbol{\Sigma} \boldsymbol{\Gamma} \boldsymbol{w}| &\ge |\boldsymbol{w}_{Q_0}^\top \boldsymbol{\Gamma}^\top \boldsymbol{\Sigma} \boldsymbol{\Gamma} \boldsymbol{w}_{Q_0}| - |\boldsymbol{w}_{Q_0}^\top \boldsymbol{\Gamma}^\top \boldsymbol{\Sigma} \boldsymbol{\Gamma} \boldsymbol{w}_{Q_0^c}| \\
&\ge \left(\sigma^P_{\min} - (s-1)\sigma \right) |\boldsymbol{w}_{Q_0}|_2^2 - \left| \sum_{a,b} \Sigma^\Gamma_{ab} w_{Q_0 a} w_{Q_0^c b} \right| \\
&\ge \left(\sigma^P_{\min} - (s-1)\sigma \right) |\boldsymbol{w}_{Q_0}|_2^2 - \sigma |\boldsymbol{w}_{Q_0}|_1 |\boldsymbol{w}_{Q_0^c}|_1 \\
&\ge \left(\sigma^P_{\min} - (s-1)\sigma \right) |\boldsymbol{w}_{Q_0}|_2^2 - \sigma |\boldsymbol{w}_{Q_0}|_1^2 \\
&\ge \left(\sigma^P_{\min} - (2s-1)\sigma \right) |\boldsymbol{w}_{Q_0}|_2^2.
\end{aligned}$$

Together with $|\boldsymbol{w}_{Q_0}^\top \boldsymbol{\Gamma}^\top \boldsymbol{\Sigma}\boldsymbol{\Gamma}\boldsymbol{w}| \le |\boldsymbol{w}_{Q_0}|_1 |\boldsymbol{\Gamma}^\top \boldsymbol{\Sigma}\boldsymbol{\Gamma}\boldsymbol{w}|_\infty \le s^{1/2}|\boldsymbol{w}_{Q_0}|_2 |\boldsymbol{\Gamma}^\top \boldsymbol{\Sigma}\boldsymbol{\Gamma}\boldsymbol{w}|_\infty$, this implies that

$$|\boldsymbol{w}|_1 \le 2s^{1/2}|\boldsymbol{w}_{Q_0}|_2 \le \frac{2s|\boldsymbol{\Gamma}^\top \boldsymbol{\Sigma}\boldsymbol{\Gamma}\boldsymbol{w}|_\infty}{\sigma_{\min}^P - (2s-1)\sigma},$$

so Equation (3.5) becomes

$$|\boldsymbol{w}|_\infty \le \frac{1}{\sigma_{\min}^P}\left\{1 + \frac{\sigma_{\min}^P}{\sigma_{\min}^P - (2s-1)\sigma}\right\}|\boldsymbol{\Gamma}^\top \boldsymbol{\Sigma}\boldsymbol{\Gamma}\boldsymbol{w}|_\infty.$$

By the feasibility of $\boldsymbol{\theta}_0$, we have

$$\begin{aligned}|\boldsymbol{\Gamma}^\top \boldsymbol{\Sigma}\boldsymbol{\Gamma}\boldsymbol{w}|_\infty &= |\boldsymbol{\Gamma}^\top \boldsymbol{\Sigma}\boldsymbol{\Gamma}\widehat{\boldsymbol{\theta}} - \boldsymbol{\Gamma}^\top \boldsymbol{s}|_\infty \\ &\le |\boldsymbol{\Gamma}^\top \widehat{\boldsymbol{S}}\boldsymbol{\Gamma}\widehat{\boldsymbol{\theta}} - \boldsymbol{\Gamma}^\top \widehat{\boldsymbol{s}}|_\infty + |\boldsymbol{\Gamma}^\top(\widehat{\boldsymbol{S}} - \boldsymbol{\Sigma})\boldsymbol{\Gamma}\widehat{\boldsymbol{\theta}}|_\infty + |\boldsymbol{\Gamma}^\top(\widehat{\boldsymbol{s}} - \boldsymbol{s})|_\infty \\ &\le \lambda_n + \|\boldsymbol{\Gamma}^\top\|_\infty |\widehat{\boldsymbol{S}} - \boldsymbol{\Sigma}|_\infty \|\boldsymbol{\Gamma}\|_1 |\boldsymbol{\theta}_0|_1 \\ &+ \|\boldsymbol{\Gamma}^\top\|_\infty \left(|\widehat{\boldsymbol{S}}^X - \boldsymbol{\Sigma}^X|_\infty + |\widehat{\boldsymbol{S}}^Y - \boldsymbol{\Sigma}^Y|_\infty\right) \\ &\le \lambda_n + C\left(\log p / \min(n_X, n_Y)\right)^{1/2} = 2\lambda_n.\end{aligned}$$

Thus

$$|\widehat{\boldsymbol{\Delta}} - \boldsymbol{\Delta}_0|_\infty = |\boldsymbol{w}|_\infty \le \frac{2}{\sigma_{\min}^P}\left\{1 + \frac{\sigma_{\min}^P}{\sigma_{\min}^P - (2s-1)\sigma}\right\}\lambda_n,$$

and this concludes Theorem 1.3.

Proof of Theorem 1.1

Let $\widehat{\delta}_{jk}^{\tau_n}$ be the (j,k)th entry of $\widehat{\boldsymbol{\Delta}}_{\tau_n}$. Then we have

$$P\left(\mathcal{M}(\widehat{\boldsymbol{\Delta}}_{\tau_n}) = \mathcal{M}(\boldsymbol{\Delta}_0)\right) = P\left(A \cap B \cap C\right),$$

where events A, B, C are respectively

$$A = \left\{\max_{j,k:\delta_{jk}^0 = 0} |\widehat{\delta}_{jk}^{\tau_n}| = 0\right\}, \quad B = \left\{\min_{j,k:\delta_{jk}^0 > 0} \widehat{\delta}_{jk}^{\tau_n} > 0\right\}, \quad C = \left\{\max_{j,k:\delta_{jk}^0 < 0} \widehat{\delta}_{jk}^{\tau_n} < 0\right\}.$$

Suppose $\delta_{jk}^0 > 0$, By Theorem 1.3, we have

$$\widehat{\delta}_{jk} = \delta_{jk}^0 - (\delta_{jk}^0 - \widehat{\delta}_{jk}) > 2\tau_n - \tau_n,$$

with probability tending to 1. Thus $\widehat{\delta}_{jk}^{\tau_n} = \widehat{\delta}_{jk} > 0$. Next suppose $\delta_{jk}^0 < 0$, then

$$\widehat{\delta}_{jk} = \delta_{jk}^0 - (\delta_{jk}^0 - \widehat{\delta}_{jk}) < -2\tau_n + \tau_n,$$

with probability tending to 1. Thus $\widehat{\delta}_{jk}^{\tau_n} = \widehat{\delta}_{jk} < 0$. Finally, for $\delta_{jk}^0 = 0$, $|\widehat{\delta}_{jk}| = |\widehat{\delta}_{jk} - \delta_{jk}^0| \le \tau_n$ with probability tending to 1, thus $\widehat{\delta}_{jk}^{\tau_n} = 0$.

3.1 Proof of Theorem 1.2

In the proof of Theorem 1.3, we obtain a result that $|\boldsymbol{w}_{Q_0^c}|_1 \leq |\boldsymbol{w}_{Q_0}|_1$. Cai et al. (2010) showed that $|\boldsymbol{w}|_2 \leq 2|\boldsymbol{w}_{Q_0 \cup Q^*}|_2$, where Q^* is the set of indices corresponding to the $s/4$ largest components of $\boldsymbol{w}_{Q_0^c}$. Then $|\boldsymbol{w}|_2 \leq 2(1.25s)^{1/2}|\boldsymbol{w}|_\infty$, and combing this with Theorem 1.3 concludes the the result.

Proof of other Theorems

For the binary data and mixed data, theoretical analysis can be conducted by the similar way as for the continuous data case due to the critical results established in Lemma 3.7 and Lemma 3.8. For saving space, we omit the proofs here.

References

Cai, T., Liu, W., Luo, X., 2011. A constrained ℓ_1 minimization approach to sparse precision matrix estimation. Journal of the American Statistical Association 106, 594–607.

Cai, T.T., Wang, L., Xu, G., 2010. Shifting inequality and recovery of sparse signals. Signal Processing, IEEE Transactions on 58, 1300–1308.

Donoho, D.L., Huo, X., 2001. Uncertainty principles and ideal atomic decomposition. Information Theory, IEEE Transactions on 47, 2845–2862.

Fan, J., Liu, H., Ning, Y., Zou, H., 2017. High dimensional semiparametric latent graphical model for mixed data. Journal of the Royal Statistical Society 79, 405–421.

Guo, J., Levina, E., Michailidis, G., Zhu, J., 2011. Joint estimation of multiple graphical models. Biometrika 98, 1–15.

Zhao, S.D., Cai, T.T., Li, H., 2014. Direct estimation of differential networks. Biometrika 101, 253–268.
